# Neutrophil to albumin ratio: a biomarker in non-alcoholic fatty liver disease and with liver fibrosis

**DOI:** 10.3389/fnut.2024.1368459

**Published:** 2024-04-08

**Authors:** Banghe Bao, Shuang Xu, Peng Sun, Liduan Zheng

**Affiliations:** ^1^Department of Pathology, Union Hospital, Tongji Medical College, Huazhong University of Science and Technology, Wuhan, China; ^2^Department of Emergency Medicine, Union Hospital, Tongji Medical College, Huazhong University of Science and Technology, Wuhan, China

**Keywords:** non-alcoholic fatty liver disease, novel inflammatory biomarker, neutrophil to albumin ratio, liver fibrosis, diagnosis

## Abstract

**Objective:**

Given the high prevalence of non-alcoholic fatty liver disease (NAFLD) and its potential to progress to liver fibrosis, it is crucial to identify the presence of NAFLD in patients to guide their subsequent management. However, the current availability of non-invasive biomarkers for NAFLD remains limited. Therefore, further investigation is needed to identify and develop non-invasive biomarkers for NAFLD.

**Methods:**

A retrospective analysis was conducted on 11,883 patients admitted to the Healthcare Center, Union Hospital, Tongji Medical College, Huazhong University of Science and Technology, from January 2016 to December 2019 and divided into NAFLD and non-NAFLD groups. Anthropometric and laboratory examination data were collected. The correlations between variables and NAFLD were evaluated using the student’s t-test or Mann–Whitney U test and binary logistic regression analysis. The predictive ability of these variables for NAFLD was assessed using the areas under the curves (AUCs) of receiver operating characteristics.

**Results:**

Among the included patients, 3,872 (32.58%) were diagnosed with NAFLD, with 386 (9.97%) individuals having liver fibrosis. Patients with NAFLD exhibited a higher proportion of males, elevated body mass index (BMI), and increased likelihood of hypertension, diabetes mellitus, and atherosclerosis. Logistic regression analysis identified the neutrophil to albumin ratio (NAR) as the most promising novel inflammation biomarkers, with the highest AUC value of 0.701, a cut-off value of 0.797, sensitivity of 69.40%, and specificity of 66.00% in identifying the risk of NAFLD. Moreover, NAR demonstrated superior predictive value in identifying NAFLD patients at risk of liver fibrosis, with an AUC value of 0.795, sensitivity of 71.30%, and specificity of 73.60% when NAR reached 1.285.

**Conclusion:**

These findings highlight that the novel inflammatory biomarker, NAR, is a convenient and easily accessible non-invasive predictor for NAFLD and NAFLD with liver fibrosis.

## 1 Introduction

In recent decades, there has been a significant increase in the prevalence of metabolic diseases, such as obesity, which not only presents inherent complications but also profoundly impacts the overall health of individuals, rendering them more susceptible to other diseases (1–3). For instance, obesity is correlated with hepatic steatosis, injury, inflammation, and fibrosis. Ludwig et al. (4) introduced the term “non-alcoholic steatohepatitis” in 1980 to characterize the association between obesity and liver-related diseases. Subsequently, the term “non-alcoholic fatty liver disease (NAFLD)” was introduced, encompassing non-alcoholic fatty liver (NAFL) and non-alcoholic steatohepatitis (NASH) subtypes. However, the term “non-alcoholic” inadequately reflects the disease’s etiology and potentially involves stigmatizing language. To address these concerns, Eslam et al. proposed the term “metabolic dysfunction-associated fatty liver disease (MAFLD)” in 2020 (5), although its widespread acceptance remains pending. In 2023, the Delphi consensus recommended renaming this condition as “metabolic dysfunction associated with steatotic liver disease (MASLD)” (6). However, the proposed new nomenclature has sparked debate (7, 8), and as a result, NAFLD continues to be used in the context of this study.

NAFLD is a progressive hepatic disorder characterized by hepatic steatosis or intracellular fat accumulation, which may progress to NASH, liver fibrosis, cirrhosis, and even hepatocellular carcinoma (HCC) (5, 9). The global prevalence of NAFLD has increased from 25.3% in 1990–2006 to 38.0% in 2016–2019. While only a tiny proportion of NAFLD patients may progress to cirrhosis or HCC, the growing NAFLD population results in an increasing number of individuals at risk of these severe outcomes (10). Despite being primarily a metabolic disorder, NAFLD encompasses various immune cell-mediated inflammatory processes, particularly during disease progression, where inflammation plays a crucial role. The diversity of hepatic immune cells in a steady state evolves during NAFLD and directly influences the severity of the disease. Importantly, NAFLD is also a significant independent risk factor for cardiovascular diseases, including atherosclerosis (11, 12). Given the complex multifactorial nature of NAFLD, its unclear etiology, its rising prevalence, and its potential to cause hepatic dysfunction and fibrosis, it has become a focal point of current research.

Recently, there has been increasing attention on the involvement of inflammatory biomarkers in the inflammatory response, including inflammatory cytokines, inflammatory cells, and platelets (13, 14). The neutrophil-to-lymphocyte ratio (NLR), platelet-to-lymphocyte ratio (PLR), platelet to neutrophil ratio (PNR), lymphocyte-to-monocyte ratio (LMR), neutrophil to albumin ratio (NAR), neutrophil percentage-to-albumin ratio (NPAR), platelet-monocyte ratio (PMR), neutrophil/lymphocyte × platelet ratio (NLPR), albumin to globulin ratio (AGR), systemic immune-inflammation index (SII), aggregate index of systemic inflammation (AISI), systemic inflammation response index (SIRI), among others, have been proposed as potential novel inflammatory biomarkers for the progression of dyslipidemia, cardiovascular and cerebrovascular diseases (15–21). A study demonstrated a significant elevation in the NLR in cases of advanced inflammation, providing valuable insights into the assessing of NAFLD severity in patients (22). Another study established a significant association between the SII and hepatic steatosis (23). Furthermore, an independent study elucidated the pivotal role of platelets in the initiating and propagating of inflammatory diseases (24). However, the relationship between these inflammatory biomarkers and NAFLD remains controversial. Some studies have shown a positive correlation, while others have shown negative or no significant associations (25–28). We believe this discrepancy may be due to bias caused by a small amount of data, thus necessitating research based on a large amount of data for support. Furthermore, liver biopsy remains the gold standard for diagnosing NAFLD or NAFLD with liver fibrosis (29). However, this procedure is invasive, expensive, and carries potential complications (30). Therefore, it is critical to develop non-invasive biomarkers that can accurately identify patients with NAFLD and even NAFLD with liver fibrosis. This study aims to assess the clinical value of novel inflammatory biomarkers in the non-invasive diagnosis of NAFLD and NAFLD with liver fibrosis.

## 2 Materials and methods

### 2.1 Patients

**Figure 1:**
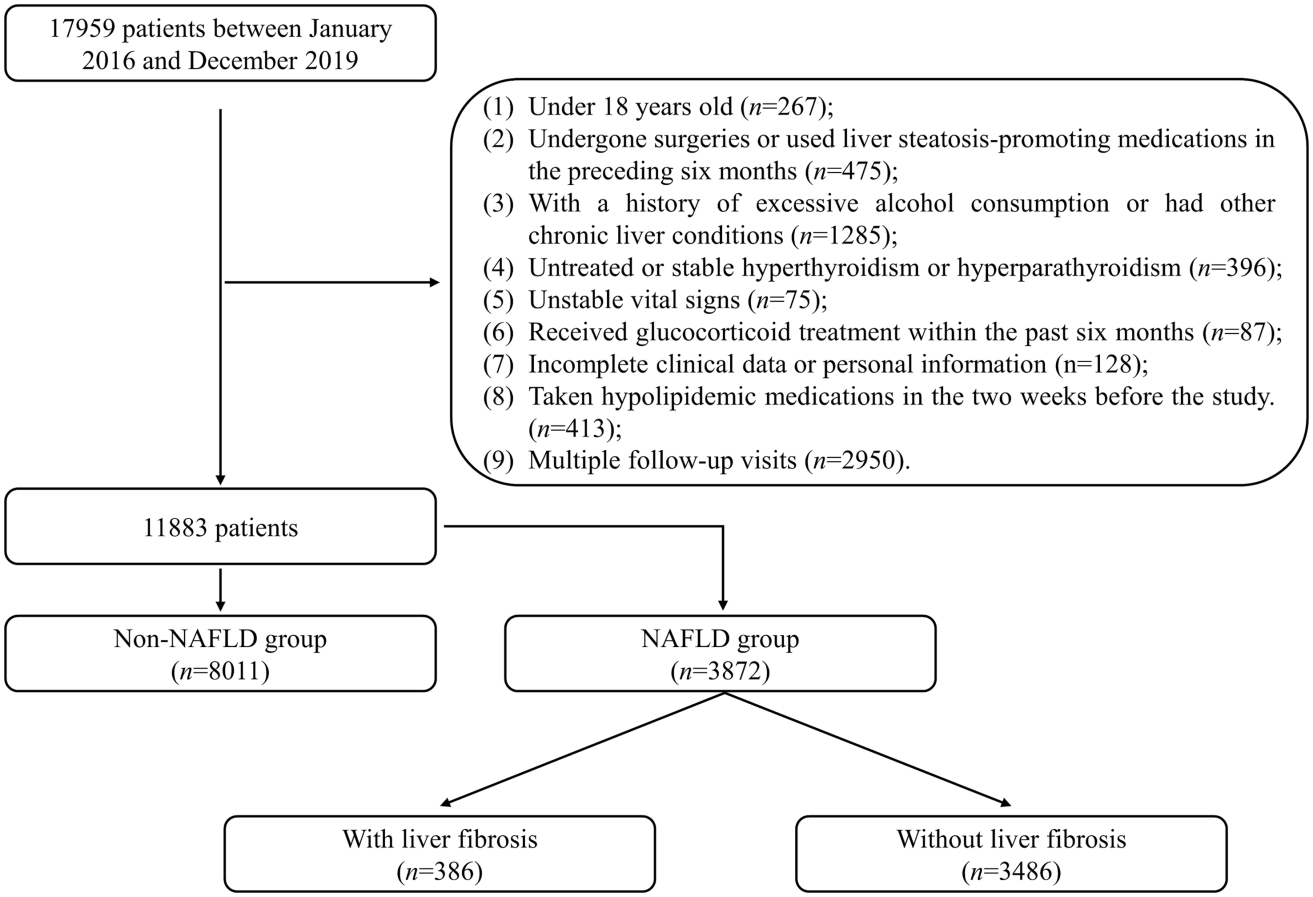
Flowchart describing the recruitment process of the study population. NAFLD, non-alcoholic fatty liver disease.

This retrospective study included 17,959 patients admitted to the Healthcare Center, Union Hospital, Tongji Medical College, Huazhong University of Science and Technology between January 2016 and December 2019 were included. Exclusion criteria were applied to ensure the study’s validity: (1) patients under the age of 18 (*n* = 267); (2) patients who had undergone surgeries or used liver steatosis-promoting medications in the preceding 6 months (*n* = 475); (3) patients with a history of excessive alcohol consumption (defined as an average daily intake exceeding 20 g for females or 30 g for males) or other chronic liver diseases (*n* = 1,285); (4) patients with untreated or stable hyperthyroidism or hyperparathyroidism (*n* = 396); (5) patients with unstable vital signs (*n* = 75); (6) patients who had received glucocorticoid treatment within the past 6 months (*n* = 87); (7) patients with incomplete clinical data or personal information (*n* = 128); (8) patients who had taken hypolipidemic medications in the 2 weeks before the study (*n* = 413). Only the initial visit was considered for patients with multiple follow-up visits (*n* = 2,950). After applying these criteria, 11,883 patients were included in the statistical analysis and divided into the NAFLD and non-NAFLD groups. The recruitment process of the study population is illustrated in Figure 1. The study was conducted in accordance with the Declaration of Helsinki (as revised in 2013). The study was approved by the Ethics Committee of Union Hospital, Tongji Medical College, Huazhong University of Science and Technology (No. 2023-0611), and individual consent for this retrospective analysis was waived.

### 2.2 Definition of NAFLD and NAFLD with fibrosis

The definition of NAFLD entails the identification of hepatic steatosis through ultrasound examination, with the exclusion of individuals who engage in excessive alcohol consumption (>20 g/day for females or >30 g/day for males), as well as those with positive serology for hepatitis B/C/D or other exogenous factors such as long-term use of steatogenic medications (31). Furthermore, the pathological examination of liver biopsy tissue was also applied to diagnose NAFLD directly.

The definition of NAFLD with liver fibrosis was established based on the pathological results of liver biopsy tissue and transient elastography (FibroScan) in patients diagnosed with NAFLD (31).

### 2.3 Clinical and laboratory data collection

Patient demographics, including age, gender, height, weight, and history of tobacco and alcohol consumption (whether current or past, all classified as a history of tobacco or alcohol consumption), were obtained from the hospital’s electronic records. Body mass index (BMI, kg/m^2^) = $\frac{\mathrm{weight}}{\mathrm{height}2}$ . Moreover, patients’ self-reported medical histories and measurements of fasting plasma glucose, systolic blood pressure (SBP, mmHg) in the right arm, and diastolic blood pressure (DBP, mmHg) were used to assess the presence of hypertension and diabetes mellitus. Upon hospital admission, hepatic and renal function were evaluated through biochemical analyses conducted using an automated biochemical analysis system (Roche Diagnostics GmbH, Mannheim, Germany). Hepatic function parameters measured included alanine aminotransferase (ALT, IU/L), aspartate aminotransferase (AST, IU/L), total protein (TP, g/L), albumin (ALB, g/L), and globulin (GLB, g/L), triglycerides (TG, mmol/L), total cholesterol (TC, mmol/L), high-density lipoprotein cholesterol (HDL-c, mmol/L), and low-density lipoprotein cholesterol (LDL-c, mmol/L). Renal function was assessed by measuring serum creatinine (SCr, μmol/L), blood urea nitrogen (BUN, μmol/L), and estimated glomerular filtration rate (eGFR, mL/min/1.73m^2^) levels. Peripheral blood cell counts were determined using an automated hematology analyzer (Beckman Coulter HMX-AL, Brea, CA, United States), including white blood cells (WBC, ×10^9^/L), neutrophil count (×10^9^/L) and percentage, lymphocyte count (×10^9^/L) and percentage, monocyte count (×10^9^/L) and percentage, hemoglobin (Hb, g/L), and platelet count (PLT, ×10^9^/L). Additionally, various ratios were calculated based on the formula:


$$PLR=\frac{\mathrm{platelet}}{\mathrm{lymphocyte}}$$

$$NLR=\frac{\mathrm{neutrophil}}{\mathrm{lymphocyte}}$$

$$LMR=\frac{\mathrm{lymphocyte}}{\mathrm{monocyte}}$$

$$PNR=\frac{\mathrm{platelet}}{\mathrm{neutrophil}}$$

$$PMR=\frac{\mathrm{platelet}}{\mathrm{monocyte}}$$

$$NAR=\frac{\mathrm{neutrophil}}{\mathrm{albumin}}\times 10$$

$$\mathrm{NPAR}=\frac{\mathrm{neutrophil}\;\mathrm{percentage}}{\mathrm{albumin}}$$

$$\mathrm{NLPR}=\frac{\mathrm{neutrophil}}{\mathrm{lymphocyte}\times \mathrm{platelet}}\times 100$$

$$AGR=\frac{\mathrm{albumin}}{\mathrm{globulin}}$$

$$SII=\frac{\mathrm{neutrophil}\times \mathrm{platelet}}{\mathrm{lymphocyte}}$$

$$\mathrm{SIRI}=\frac{\mathrm{neutrophil}\times \mathrm{monocyte}}{\mathrm{lymphocyte}}$$

$$\mathrm{AISI}=\frac{\mathrm{neutrophil}\times \mathrm{platelet}\times \mathrm{monocyte}}{\mathrm{lymphocyte}}$$

### 2.4 Statistical analysis

Statistical analysis was conducted by presenting continuous variables as mean (standard deviation, SD) or median (interquartile range, IQR), depending on their distribution normality, which was assessed using the Kolmogorov–Smirnov test. Student’s *t*-test or Mann–Whitney *U* test was used to compare the difference between continuous variables when appropriate. During data analysis, extreme values within the sample data will be excluded. Categorical variables were presented as count (proportion), and the difference between categorical variables was assessed using the Chi-square test or Fisher’s exact test. Logistic regression analysis was conducted to evaluate the correlation between novel inflammation biomarkers and NAFLD, while adjusting for age, sex, BMI, serum biochemical indexes, clinical diagnosis, and other relevant factors. Receiver operating characteristic (ROC) curves were subsequently generated to calculate the area under the curves (AUCs) for each novel inflammation biomarker in identifying NAFLD and its associated liver fibrosis. The optimal cutoff value, sensitivity, and specificity were determined using the Youden index. Statistical analyses were conducted using SPSS software, version 24.0 (IBM Corp., United States). *p* < 0.05 was considered statistically significant.

## 3 Results

### 3.1 Study populations

A total of 11,883 individuals were included in this study, comprising 7,529 (63.36%) males and 4,354 (36.64%) females. The mean age of the participants was 47.49 ± 15.42 years old, and the mean BMI was 23.99 ± 3.26 kg/m^2^. Among the included individuals, 23.11% had a history of hypertension, 5.39% had diabetes mellitus, and 9.17% had atherosclerosis. Tobacco consumption was reported by 24.08% of the participants, while alcohol consumption was reported by 11.40% (Table 1).

**Table 1:** Clinical and laboratory characteristics of patients between NAFLD and non-NAFLD groups.

Variables	Total (*n* = 11,883)	Non-NAFLD (*n* = 8,011)	NAFLD (*n* = 3,872)	*p*-value
*Demographics variables*				
Age (year)	47.49 ± 15.42	47.57 ± 15.47	46.79 ± 14.95	0.110
Gender (n, %)				<0.001
Male	7,529 (63.36%)	4,457 (55.64%)	3,072 (79.34%)	
Female	4,354 (36.64%)	3,554 (44.36%)	800 (20.66%)	
BMI (kg/m^2^)	23.99 ± 3.26	23.72 ± 3.08	26.25 ± 3.85	<0.001
Hypertension (*n*, %)	2,746 (23.11%)	1,364 (17.03%)	1,382 (35.69%)	<0.001
Smoking (*n*, %)	2,862 (24.08%)	1940 (24.22%)	922 (23.81%)	0.785
Diabetes mellitus (*n*, %)	641 (5.39%)	298 (3.70%)	343 (8.90%)	<0.001
Drinking (*n*, %)	1,355 (11.40%)	945 (11.80%)	410 (10.60%)	0.272
Atherosclerosis (*n*, %)	1,090 (9.17%)	638 (7.96%)	452 (11.67%)	<0.001
*Laboratory variables*				
WBC (×10^9^/L)	6.16 ± 1.65	6.03 ± 1.40	7.31 ± 2.77	<0.001
Neutrophil percentage	57.36 ± 7.9	57.06 ± 7.82	59.94 ± 8.14	<0.001
Neutrophil (×10^9^/L)	3.56 ± 1.12	3.47 ± 1.06	4.39 ± 1.28	<0.001
Lymphocyte percentage	33.67 ± 7.58	33.95 ± 7.51	31.29 ± 7.77	<0.001
Lymphocyte (×10^9^/L)	2.05 ± 0.86	2.02 ± 0.57	2.28 ± 0.60	<0.001
Monocyte percentage	6.06 ± 1.59	6.12 ± 1.60	6.12 ± 1.57	0.927
Monocyte (×10^9^/L)	0.37 ± 0.14	0.36 ± 0.12	0.44 ± 0.27	<0.001
Hb (g/L)	146.32 ± 14.52	145.85 ± 14.55	150.43 ± 13.65	<0.001
PLT (×10^9^/L)	230.23 ± 56.03	228.70 ± 55.77	243.43 ± 56.58	<0.001
TP (g/L)	74.71 ± 4.21	74.67 ± 4.19	75.02 ± 4.31	0.008
ALB (g/L)	47.02 ± 2.61	47.02 ± 2.60	47.06 ± 2.67	0.562
GLB (g/L)	27.69 ± 3.44	27.66 ± 3.44	27.96 ± 3.44	0.005
ALT (IU/L)	25.68 ± 19.32	24.30 ± 17.60	37.68 ± 27.60	<0.001
AST (IU/L)	23.52 ± 11.95	22.97 ± 11.40	28.21 ± 15.19	<0.001
TG (mmol/L)	1.63 ± 1.34	1.56 ± 1.24	2.26 ± 1.84	<0.001
TC (mmol/L)	4.78 ± 0.91	4.76 ± 0.91	4.90 ± 0.92	<0.001
HDL-c (mmol/L)	1.40 ± 0.34	1.41 ± 0.34	1.26 ± 0.30	<0.001
LDL-c (mmol/L)	2.76 ± 0.74	2.75 ± 0.74	2.84 ± 0.76	<0.001
BUN (μmol/L)	4.92 ± 1.38	4.93 ± 1.39	4.88 ± 1.30	0.238
SCr (μmol/L)	73.32 ± 19.69	73.17 ± 20.09	74.63 ± 15.69	0.054
eGFR (mL/min/1.73 m^2^)	105.94 ± 23.67	106.05 ± 23.73	105.07 ± 23.08	0.190

NAFLD, non-alcoholic fatty liver disease; BMI, body mass index; WBC, white blood cell; Hb, hemoglobin; PLT, platelet; TP, total protein; ALB, albumin; GLB, globulin; ALT, alanine transaminase; AST, aspartate aminotransferase; TG, triglyceride; TC, total cholesterol; HDL-c, high density lipoprotein cholesterol; LDL-c, low-density lipoprotein cholesterol; BUN, blood urea nitrogen; SCr, serum creatinine; eGFR, estimated glomerular filtration rate.

### 3.2 Comparison between NAFLD and non-NAFLD groups

Among the total of 11,883 individuals included in the study, 3,872 (32.58%) were diagnosed with NAFLD, and 386 NAFLD individuals (9.97%) progressed to liver fibrosis. NAFLD group has a higher proportion of males (79.34% vs. 55.64%, *p* < 0.001) and a higher BMI (26.25 ± 3.85 vs. 23.72 ± 3.08, *p* < 0.001) compared to the non-NAFLD group. Patients with NAFLD were also more likely to have hypertension (35.69% vs. 17.03%, *p* < 0.001), diabetes mellitus (8.90% vs. 3.70%, *p* < 0.001), and atherosclerosis (11.67% vs. 7.96%, *p* < 0.001) compared to the patients with non-NAFLD. However, there were no significant differences in terms of age (46.79 ± 14.95 vs. 47.57 ± 15.47, *p* = 0.11), tobacco use (23.81% vs. 24.22%, *p* = 0.785), and alcohol consumption (10.60% vs. 11.80%, *p* = 0.272) between the NAFLD and non-NAFLD groups. Regarding laboratory testing, the NAFLD group exhibited significantly elevated levels of traditional inflammation biomarkers (*p* < 0.05), including WBC, neutrophil percentage and count, lymphocyte percentage and count, and monocyte count (with no significant difference in monocyte percentage, *p* > 0.05). Hb and PLT also showed significant differences compared to the non-NAFLD group (*p* < 0.05). Liver function indicators, including TP, ALT, AST, TG, TC, HDL-c, and LDL-c, exhibited significant differences compared to the non-NAFLD group (*p* < 0.05). However, the two groups had no significant differences in renal function indicators such as BUN, Cr, and eGFR (*p* > 0.05) (Table 1).

**Figure 2:**
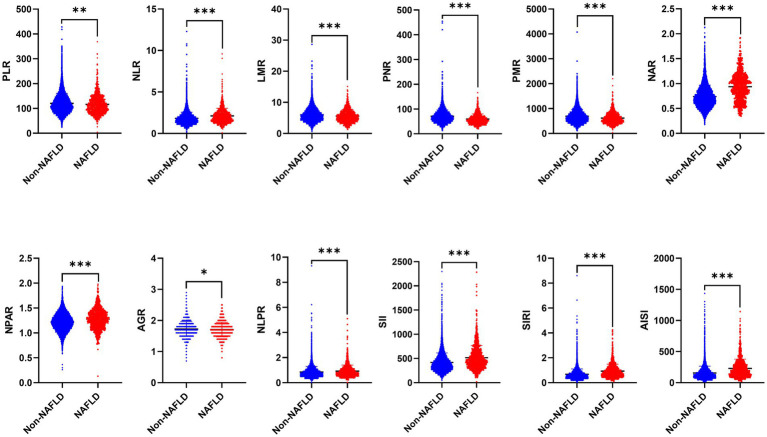
The distribution of 12 novel inflammatory biomarkers in patients between NAFLD and non-NAFLD groups. NAFLD, non-alcoholic fatty liver disease; PLR, platelet to lymphocyte ratio; NLR, neutrophil to lymphocyte ratio; LMR, lymphocyte to monocyte ratio; PNR, platelet to neutrophil ratio; PMR, platelet-monocyte ratio; NAR, neutrophil to albumin ratio; NPAR, neutrophil percentage-to-albumin ratio; NLPR, neutrophil/lymphocyte × platelet ratio; AGR, albumin to globulin ratio; SII, systemic immune-inflammation index; SIRI, systemic inflammation response index; AISI, aggregate index of systemic inflammation.

As mentioned, there are significant differences in traditional inflammatory biomarkers between the NAFLD and non-NAFLD groups. Additionally, our analysis also revealed significant differences in all novel inflammation biomarkers, including PLR, NLR, LMR, PNR, PMR, NAR, NPAR, NLPR, AGR, SII, SIRI, and AISI, between the NAFLD and non-NAFLD groups (*p* < 0.05) (Table 2 and Figure 2).

**Table 2:** Novel inflammatory biomarker characteristics of patients between NAFLD and non-NAFLD groups.

Variables	Total (*n* = 11,883)	Non-NAFLD (*n* = 8,011)	NAFLD (*n* = 3,872)	*p*-value
PLR	119.98 ± 39.66	120.38 ± 39.71	116.49 ± 39.07	0.002
NLR	1.86 ± 0.77	1.83 ± 0.74	2.12 ± 0.93	<0.001
LMR	5.96 ± 2.16	6.00 ± 2.17	5.60 ± 2.04	<0.001
PNR	69.93 ± 25.69	71.11 ± 25.94	59.68 ± 20.81	<0.001
PMR	686.59 ± 273.36	694.23 ± 276.18	620.47 ± 237.70	<0.001
NAR	0.76 ± 0.24	0.74 ± 0.23	0.94 ± 0.28	<0.001
NPAR	1.22 ± 0.18	1.22 ± 0.18	1.28 ± 0.20	<0.001
NLPR	0.86 ± 0.45	0.85 ± 0.45	0.92 ± 0.49	<0.001
AGR	1.73 ± 0.24	1.73 ± 0.25	1.71 ± 0.23	0.010
SII	427.58 ± 204.96	417.36 ± 196.21	516.14 ± 252.41	<0.001
SIRI	0.71 ± 0.45	0.68 ± 0.43	0.93 ± 0.55	<0.001
AISI	163.99 ± 115.57	156.48 ± 108.64	229.01 ± 148.72	<0.001

NAFLD, non-alcoholic fatty liver disease; PLR, platelet to lymphocyte ratio; NLR, neutrophil to lymphocyte ratio; LMR, lymphocyte to monocyte ratio; PNR, platelet to neutrophil ratio; PMR, platelet-monocyte ratio; NAR, neutrophil to albumin ratio; NPAR, neutrophil percentage-to-albumin ratio; NLPR, neutrophil/lymphocyte × platelet ratio; AGR, albumin to globulin ratio; SII, systemic immune-inflammation index; SIRI, systemic inflammation response index; AISI, aggregate index of systemic inflammation.

### 3.3 Binary logistic regression analysis of NAFLD

The novel inflammation biomarkers, including PLR, NLR, LMR, PNR, PMR, NAR, NPAR, NLPR, AGR, SII, SIRI, and AISI, were significantly associated with the risk of NAFLD. After adjusting for various potential confounding factors such as age, gender, BMI, and others, the novel inflammation biomarkers maintained statistical significance, except PLR and AGR (Table 3).

**Table 3:** Logistic regression analysis of novel inflammation biomarkers to predict the risk of NAFLD.

Variables	Crude model			Model 1			Model 2		
	OR	95% CI	*p*-value	OR	95% CI	*p*-value	OR	95% CI	*p*-value
PLR	0.997	0.996–0.999	0.002	1.000	0.998–1.001	0.681	1.001	0.999–1.002	0.518
NLR	1.479	1.382–1.583	<0.001	1.583	1.470–1.706	<0.001	1.620	1.500–1.749	<0.001
LMR	0.909	0.881–0.938	<0.001	0.912	0.882–0.943	<0.001	0.902	0.872–0.934	<0.001
PNR	0.977	0.974–0.98	<0.001	0.978	0.974–0.981	<0.001	0.978	0.975–0.982	<0.001
PMR	0.999	0.999–0.999	<0.001	0.999	0.999–0.999	<0.001	0.999	0.999–0.999	<0.001
NAR	17.720	14.021–22.396	<0.001	14.087	10.964–18.1	<0.001	14.082	10.899–18.194	<0.001
NPAR	6.205	4.431–8.688	<0.001	8.779	6.024–12.796	<0.001	10.869	7.328–16.121	<0.001
NLPR	1.323	1.176–1.488	<0.001	1.478	1.294–1.687	<0.001	1.560	1.359–1.79	<0.001
AGR	0.711	0.548–0.922	0.01	0.633	0.477–0.841	0.002	0.745	0.557–0.996	0.061
SII	1.002	1.002–1.002	<0.001	1.002	1.002–1.002	<0.001	1.002	1.002–1.002	<0.001
SIRI	2.498	2.232–2.796	<0.001	2.438	2.159–2.753	<0.001	2.452	2.163–2.779	<0.001
AISI	1.004	1.003–1.004	<0.001	1.003	1.003–1.004	<0.001	1.003	1.003–1.004	<0.001

Crude model adjusted for none. Model 1 adjusted for age, gender, and BMI. Model 2 adjusted for age, gender, and BMI, hypertension, diabetes mellitus, smoking, drinking, atherosclerosis, Hb, PLT, ALT, AST, TG, TC, HDL-c, LDL-c, BUN, SCr, eGFR. NAFLD, non-alcoholic fatty liver disease; OR, odds ratio; CI, confidential interval; BMI, body mass index; Hb, hemoglobin; PLT, platelet; ALT, alanine transaminase; AST, aspartate aminotransferase; TG, triglyceride; TC, total cholesterol; HDL-c, high density lipoprotein cholesterol; LDL-c, low-density lipoprotein cholesterol; BUN, blood urea nitrogen; SCr, serum creatinine; eGFR, estimated glomerular filtration rate. PLR, platelet to lymphocyte ratio; NLR, neutrophil to lymphocyte ratio; LMR, lymphocyte to monocyte ratio; PNR, platelet to neutrophil ratio; PMR, platelet-monocyte ratio; NAR, neutrophil to albumin ratio; NPAR, neutrophil percentage-to-albumin ratio; NLPR, neutrophil/lymphocyte × platelet ratio; AGR, albumin to globulin ratio; SII, systemic immune-inflammation index; SIRI, systemic inflammation response index; AISI, aggregate index of systemic inflammation.

### 3.4 Evaluation of the efficacy of various novel inflammation biomarkers in predicting NAFLD by ROC curve

**Figure 3:**
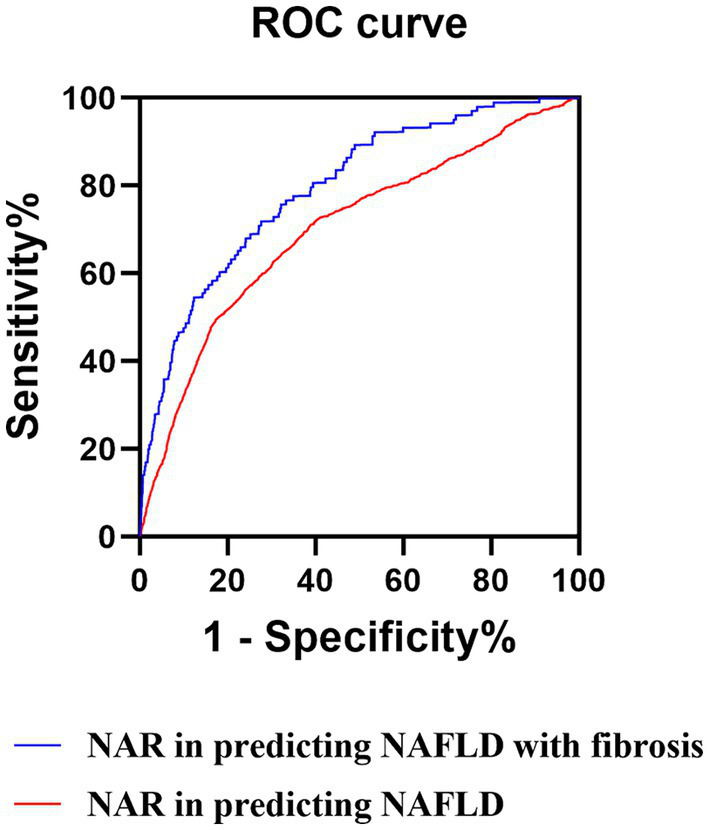
ROC curves of the NAR. ROC, receiver operator characteristic; NAR, neutrophil to albumin ratio; NAFLD, non-alcoholic fatty liver disease.

We utilized ROC curve analysis to evaluate the predictive accuracy of those above potential novel inflammation biomarkers for NAFLD. The results demonstrated that the predictive value of NAR for NAFLD was higher compared to that of NLR, LMR, PNR, PMR, NPAR, NLPR, SII, SIRI, and AISI, with an AUC value of 0.701 (95% CI: 0.694–0.708, sensitivity: 69.40%, specificity: 66.00%) (Table 4 and Figure 3).

**Table 4:** AUCs of novel inflammation biomarkers in predicting NAFLD.

Variables	AUC (95% CI)	Sensitivity	Specificity	Youden index	Cut-off
NLR	0.605 (0.588–0.623)	0.523	0.642	0.165	1.923
LMR	0.546 (0.534–0.557)	1.000	0.001	0.001	1.272
PNR	0.637 (0.627–0.644)	1.000	0.002	0.002	20.080
PMR	0.565 (0.554–0.576)	0.994	0.007	0.001	234.504
NAR	0.701 (0.694–0.708)	0.694	0.660	0.354	0.798
NPAR	0.588 (0.580–0.596)	0.531	0.612	0.143	1.265
NLPR	0.545 (0.537–0.552)	0.390	0.680	0.070	0.927
SII	0.628 (0.621–0.636)	0.583	0.624	0.207	430.591
SIRI	0.657 (0.650–0.665)	0.547	0.705	0.252	0.768
AISI	0.670 (0.663–0.678)	0.570	0.700	0.270	176.115

AUC, areas under the curves; NAFLD, non-alcoholic fatty liver disease; NLR, neutrophil to lymphocyte ratio; LMR, lymphocyte to monocyte ratio; PNR, platelet to neutrophil ratio; PMR, platelet-monocyte ratio; NAR, neutrophil to albumin ratio; NPAR, neutrophil percentage-to-albumin ratio; NLPR, neutrophil/lymphocyte × platelet ratio; SII, systemic immune-inflammation index; SIRI, systemic inflammation response index; AISI, aggregate index of systemic inflammation.

### 3.5 Evaluation of the efficacy of NAR in predicting NAFLD with liver fibrosis by ROC curve

We also evaluate the accuracy of those above potential novel inflammation biomarkers in predicting NAFLD with liver fibrosis by utilizing the ROC curve. The results revealed that NAR had an AUC value of 0.795 (95% CI: 0.785–0.805, sensitivity: 71.3%, specificity: 73.6%) with a cut-off value 1.285 (Table 5; Supplementary Table S1; Figure 3). Additionally, we calculated the fibrosis-4 (FIB-4), AST to platelet ratio index (APRI), and AST/ALT ratio, commonly used non-invasive indicators for predicting NAFLD with liver fibrosis, and conducted ROC curve analysis. The results indicated that FIB-4, APRI, and the AST/ALT ratio exhibited limited predictive capability for NAFLD with liver fibrosis in comparison to NAR, as evidenced by their respective AUC values of 0.535 (95% CI: 0.527–0.543), 0.517 (95% CI: 0.505–0.528), and 0.532 (95% CI: 0.508–0.555) (Supplementary Table S2). Therefore, NAR can also be a promising predictor for NAFLD with liver fibrosis.

**Table 5:** AUC of the NAR in predicting NAFLD with liver fibrosis.

Variable	AUC (95% CI)	Sensitivity	Specificity	Youden index	Cut-off
NAR	0.795 (0.785–0.805)	0.713	0.736	0.449	1.285

AUC, areas under the curves; NAR, neutrophil to albumin ratio; NAFLD, non-alcoholic fatty liver disease; CI, confidential interval.

## 4 Discussion

NAFLD is characterized by hepatic fat accumulation, which can progress to liver fibrosis and HCC. Moreover, the incidence rate of NAFLD is also rising rapidly. Therefore, it is crucial not only to diagnose NAFLD but also to identify NAFLD with liver fibrosis. In this retrospective study, the incidence rate of NAFLD was 32.58%, with 9.97% of cases accompanied by liver fibrosis. The pathogenesis of NAFLD is complex, with inflammation playing a crucial role in its development and progression (32). Immune cells in the liver microenvironment can influence the onset and severity of the disease (9). Neutrophil accumulation is an early event in mouse models of NAFLD (33–35). Depletion of neutrophils has been shown to reduce serum ALT activity, liver inflammation, and mRNA levels of proinflammatory genes in the early stage of NAFLD. However, this effect diminishes as NAFLD progresses (33). Neutrophils appear to contribute to the early development of NAFLD by forming neutrophil extracellular traps (NETs), but their contribution to the later stages of the disease remains unclear. Serum albumin, a major plasma protein synthesized in the liver, has been associated with specific inflammatory mediators. Low albumin levels may lead to adverse outcomes by disrupting bodily fluid distribution (36, 37). The predictive value of albumin in reflecting inflammation or its independent role is still uncertain. In this study, we compared inflammatory biomarkers between two groups and found that traditional inflammation biomarkers were positively correlated with NAFLD. Furthermore, novel inflammation biomarkers such as NLR, NAR, NPAR, NLPR, SII, SIRI, and AISI were also positively correlated with NAFLD, while LMR, PNR, and PMR showed negative correlations. These findings highlight the diagnostic significance of novel inflammatory biomarkers in identifying NAFLD. Notably, NAR emerged as the most significant risk factor for predicting both NAFLD and NAFLD with liver fibrosis, showing a positive correlation between the severity of NAR and the various stages of NAFLD.

While various diagnostic modalities are available for assessing liver fibrosis, such as liver biopsy and transient elastography (FibroScan) utilized in this study, Though liver biopsy remains the reference standard for diagnosing NAFLD or NAFLD with liver fibrosis (29), it is essential to note that liver biopsy is an invasive, costly procedure (30). Moreover, despite the FibroScan recommended by the European Association for the Study of the Liver (EASL) and the American Association for the Study of Liver Diseases (AASLD) to assess liver fibrosis in NAFLD (31), its accuracy may be compromised by factors such as obesity and challenges in avoiding interference from blood vessels, bile ducts, and ascites (38, 39). Moreover, limited resources in many developing countries hinder routine FibroScan utilization for detecting NAFLD and fibrotic liver conditions. Our data primarily stem from individuals undergoing physical examinations, and given the relatively high cost of FibroScan and liver biopsy, their applications are not used for routine physical examinations of individuals. Consequently, many individuals are only tested for liver fibrosis after an NAFLD diagnosis. Therefore, continuous exploration of non-invasive predictive biomarkers for NAFLD and associated fibrosis is imperative. NAR, derived directly from hematology examination results, offers a convenient and cost-effective means to predict NAFLD and NAFLD with fibrosis. In addition to FibroScan, other non-invasive indicators such as FIB-4, APRI, and AST/ALT ratio are employed for liver fibrosis prediction. However, their predictive efficacy varies across diverse populations. A study on Iranians suggests that FIB-4 and APRI effectively predict liver fibrosis, while the AST/ALT ratio exhibits lower effectiveness (40). Conversely, investigations in India indicate that FIB-4, APRI, and the AST/ALT ratio have limited predictive value for liver fibrosis, with respective AUCs of 0.60 (95% CI: 0.54–0.65), 0.68 (95% CI: 0.62–0.73), and 0.58 (95% CI: 0.53–0.64) (41). Our results also indicate that FIB-4, APRI, and the AST/ALT ratio exhibit limited predictive capability for NAFLD with liver fibrosis. There was a study has also indicated that FIB-4 ≥ 1.3 results in false positives in 35% of patients (42). Another study reported an AUC of 0.810 (95% CI: 0.794–0.825) for NPAR in predicting NAFLD, and it was also associated with an increased risk of advanced fibrosis (43), which seems more superior predictive performance than NAR, as the AUC for NAR in our study was 0.701 (95% CI: 0.694–0.708). However, our study conducted a direct comparative analysis of the predictive capabilities of NAR and NPAR in forecasting NAFLD and NAFLD with liver fibrosis, highlighting the exceptional performance of NAR in these contexts. Additionally, accurate cutoff values are crucial for predicting NAFLD and biomarkers associated with liver fibrosis. The absence of specific cutoff values in previous studies has limited their clinical utility.

Our findings revealed that factors such as male gender, BMI, hypertension, diabetes mellitus, atherosclerosis, Hb, PLT, TP, GLB, ALT, AST, TG, TC, and LDL-c were positively correlated with NAFLD. Conversely, factors such as female gender and HDL-c showed a negative correlation with NAFLD, consistent with the well-established association of NAFLD with obesity, diabetes, atherogenic dyslipidemia, and arterial hypertension (11, 44). The ratio of TG to HDL-c has been identified as a surrogate biomarker for insulin resistance and can better predict metabolic syndrome and NAFLD (45). HDL-c, possessing anti-inflammatory properties, may also be crucial in preventing other inflammatory diseases (46, 47). For example, HDL-c can induce an anti-inflammatory response in macrophages through cholesterol efflux-mediated mechanisms (48). Our study found that the NAFLD group had a higher percentage of male gender (79.3%) than female gender (20.7%), indicating that male gender has a higher risk of developing NAFLD than female, consistent with previous research (49). Platelets are shown to promote hepatic steatosis, inflammation, and injury in both the early and late stages of NAFLD (32). It can also facilitate the accumulation of inflammatory cells in the liver during NAFLD in a glycoprotein Ibα-dependent manner (32). Additionally, evidence links blood components, such as Hb, to the presence and severity of NAFLD (50). Diabetes mellitus is also a metabolic disease associated with increased reactive oxygen species (ROS) levels, once ROS levels are elevated, they can trigger hyperglycemia-induced inflammatory reactions (51). Hence, diabetes may exhibit a positive correlation with NAFLD (52), Nevertheless, caution is warranted concerning the potential influence of diabetes-induced inflammatory reactions on NAR.

Limitations of this study include (1) the sample size of participants in the NAFLD with liver fibrosis group is relatively small. (2) Sole reliance on data derived from patient health examinations, leading to a need for more specific information regarding NAFLD-related HCC patients. NAFLD is increasingly acknowledged as a predominant etiology of HCC in nations such as the United States, France, and the United Kingdom (53). Notably, HCC can arise in individuals with NAFLD, even in the absence of cirrhosis. The incidence of HCC in noncirrhotic NAFLD patients is estimated to vary from 0.1 to 1.3 per 1,000 patient-years (53). Although the incidence of NAFLD-related HCC is lower compared to other etiologies, such as hepatitis C-induced HCC, the high prevalence of NAFLD underscores the critical need for immediate and comprehensive actions to enhance global awareness and address metabolic risk factors to mitigate the escalating burden of NAFLD-associated HCC. (3) The study’s cohort exclusively comprised Asian participants, potentially constraining the generalizability of these findings to other demographic groups.

## 5 Conclusion

These findings underscore the significant potential of the novel inflammatory biomarker, NAR, as a highly promising non-invasive predictor for both NAFLD and NAFLD with liver fibrosis.

## Data availability statement

The raw data supporting the conclusions of this article will be made available by the authors, without undue reservation.

## Ethics statement

The studies involving humans were approved by Ethics Committee of Union Hospital, Tongji Medical College, Huazhong University of Science and Technology. The studies were conducted in accordance with the local legislation and institutional requirements. The participants provided their written informed consent to participate in this study.

## Author contributions

BB: Writing – original draft, Writing – review & editing. SX: Writing – original draft, Writing – review & editing. PS: Project administration, Supervision, Visualization, Writing – review & editing. LZ: Project administration, Supervision, Visualization, Writing – review & editing.
