# Supplementary material for: Neutrophil to albumin ratio: a biomarker in non-alcoholic fatty liver disease and with liver fibrosis
Source: Front Nutr. 2024 Apr 8;11:1368459. doi: 10.3389/fnut.2024.1368459 (PMC11033504; doi:10.3389/fnut.2024.1368459)
Supplement: Supplementary file 1 [file Table_1.docx]

**Supplement Table S1. AUCs of novel inflammation biomarkers in predicting NAFLD with fibrosis.**

| Variables | AUC (95%CI) | Sensitivity | Specificity | Youden index | Cut-off |
| --- | --- | --- | --- | --- | --- |
| NLR | 0.586 (0.528-0.645) | 0.310 | 0.868 | 0.178 | 2.578 |
| LMR | 0.677 (0.623-0.631) | 1.000 | 0.002 | 0.002 | 1.538 |
| PNR | 0.770 (0.725-0.815) | 1.000 | 0.001 | 0.001 | 17.698 |
| PMR | 0.697 (0.644-0.749) | 1.000 | 0.003 | 0.003 | 73.291 |
| NPAR | 0.698(0.632-0.752) | 0.621 | 0.731 | 0.352 | 1.450 |
| NLPR | 0.706 (0.664-0.747) | 0.726 | 0.764 | 0.490 | 1.039 |
| SII | 0.689 (0.636-0.742) | 1.000 | 0.001 | 0.207 | 78.658 |
| SIRI | 0.576 (0.519-0.634) | 0.345 | 0.798 | 0.143 | 0.942 |
| AISI | 0.624 (0.668-0.680) | 1.000 | 0.001 | 0.001 | 10.738 |

AUC, areas under the curves; NAFLD, non-alcoholic fatty liver disease; NLR, neutrophil to lymphocyte ratio; LMR, lymphocyte to monocyte ratio; PNR, platelet to neutrophil ratio; PMR, platelet-monocyte ratio; NPAR, neutrophil percentage-to-albumin ratio; NLPR, neutrophil/lymphocyte × platelet ratio; SII, systemic immune-inflammation index; SIRI, systemic inflammation response index; AISI, aggregate index of systemic inflammation.

**Supplement Table S2. AUCs of FIB-4, APRI, and AST/ALT ratio in predicting NAFLD with fibrosis**

| Variable | AUC (95%CI) | Sensitivity | Specificity | Youden index | Cut-off |
| --- | --- | --- | --- | --- | --- |
| FIB-4 | 0.535 (0.527-0.543) | 0.717 | 0.387 | 0.104 | 1.622 |
| APRI | 0.517 (0.505-0.528) | 0.487 | 0.604 | 0.091 | 0.749 |
| AST/ALT | 0.532 (0.508-0.555) | 0.363 | 0.686 | 0.049 | 1.229 |

AUC, areas under the curves; FIB-4, fibrosis-4; APRI, AST to platelet ratio index; ALT, alanine transaminase; AST, aspartate aminotransferase; NAFLD, non-alcoholic fatty liver disease; CI, confidential interval.
